# Sonic Hedgehog: a lipid speciated hormone?

**DOI:** 10.18632/oncotarget.4969

**Published:** 2015-07-21

**Authors:** Jun Long, Emily Winterbottom, David J. Robbins

**Affiliations:** University of Miami Miller School of Medicine, Miami, Florida, USA

**Keywords:** Sonic Hedgehog, lipid modification, hormone, metabolism

In the past two decades, the Hedgehog (HH) family of secreted proteins has been demonstrated to play an essential role in both animal development and various human diseases [1]. Sonic Hedgehog (SHH) is the most extensively studied HH protein biochemically. Fully processed SHH (SHH-Np) was previously reported to be post-translationally modified by cholesterol at its carboxyl terminus and palmitic acid at its amino terminus. This dual lipid modification was shown to dictate the biological functions of SHH, including its potency, intracellular trafficking and ability to move far from its site of synthesis. However, our recent work suggests a novel addition to this well-established model, by identifying a family of lipid speciated SHH proteins that are modified at their amino termini not only by palmitic acid, but by a spectrum of different fatty acids, and that display distinct biological properties [2].

Using tissue culture cells expressing different levels of *SHH*, we observed that SHH potency was substantially higher when expressed at physiologically relevant levels than when overexpressed. We rationalized that these differences in activity might result from alterations in some post-translational modification of SHH. We therefore developed a modified purification protocol to isolate SHH from cells expressing endogenous-like levels of *SHH*, and subjected peptides from this purified SHH protein to microcapillary liquid chromatography-tandem mass spectroscopy. This analysis demonstrated that SHH-Np was heterogeneously modified at a highly conserved amino-terminal Cys25 residue, by distinct fatty acids that varied in terms of their length and saturation, as well by as a number of currently unidentified moieties. The spectrum of these modifications was dynamic, as the types of fatty acid and their degree of saturation were modulated upon changes in the growth conditions of the cells or the level of *SHH* expression. When *SHH* was expressed at low levels, 30% of SHH-Np was modified with unsaturated fatty acids, and this percentage was substantially increased upon *SHH* overexpression. Upon serum deprivation, SHH-Np was predominantly modified by unsaturated fatty acids. These results suggest that the fatty acid modification of SHH-Np is modulated in response to changes in the growth/metabolic environment of the cells producing it.

**Figure 1:**
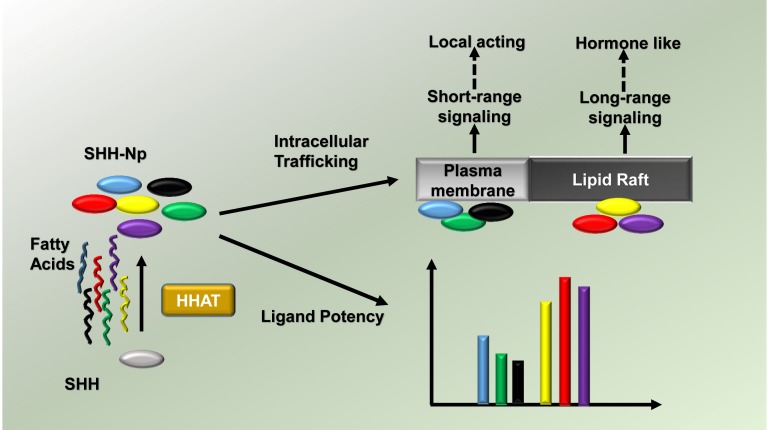
Proposed model for the role of lipid speciation in SHH function Lipid speciated SHH-Np undergoes differential intracellular trafficking to conduct short- and long-range signaling, in which its different lipid modifications also modulate its potency.

Functionally, lipid incubation studies *in vitro,* and *ex vivo* in dissected chicken embryonic limb bud tissue, indicated that SHH-Np modified with unsaturated fatty acids undergoes altered intracellular trafficking to lipid rafts and has reduced potency. Lipid rafts are wellcharacterized plasma membrane niches where SHH-Np is known to enrich, and from where it is likely secreted in a long-range acting form [3]. Thus, a simple change in the degree of fatty acid saturation could alter SHH-Np trafficking, perhaps altering the range at which it can act (Fig. 1). How these differential forms of SHH-Np might arise is not yet clear. One possible explanation is that HH acyltransferase, an enzyme that catalyzes fatty acylation of HH proteins, is able to utilize a spectrum of fatty acids as substrates [4]. Perturbation of lipid metabolism may lead to fluxes in lipid availability, thereby allowing HHAT to generate a dynamic profile of SHH post-translational modification.

Interestingly, *Drosophila* HH can act in a hormone-like manner to sense nutrient availability and accordingly modulate lipid metabolism [5]. Specifically, starvation induces elevation of a lipoprotein-associated form of circulating HH, which then triggers triacylglycerol mobilization. SHH signaling can also regulate glucose and fat metabolism in mammals, via both canonical and non-canonical signaling pathways [6]. Further, similar lipoprotein-associated Hedgehog family proteins are found in serum [7]. Therefore, we speculate that certain lipid speciated forms of SHH-Np might also function in a hormone-like manner. In such a model, these forms of SHH-Np would be produced in response to fluctuations in lipid metabolism, and would then preferentially act to modulate metabolic pathways (Figure 1).
